# Efficacy of perampanel as an adjunctive therapy in pediatric focal epilepsy

**DOI:** 10.1007/s12519-022-00678-3

**Published:** 2023-02-07

**Authors:** Wei-Ran Zhang, Liu Liu, Lu Xu, Yi Hua, Xiao-Jun Su, Pei-Fang Jiang, Zhe-Feng Yuan, Feng Gao

**Affiliations:** Department of Neurology, Children’s Hospital, Zhejiang University School of Medicine, Hangzhou 310052, China

Epilepsy is the most common neurological disease, affecting approximately 50 million people worldwide [1]. Many novel antiepileptic drugs (AEDs) displaying diverse mechanisms of action have been introduced [2]. Although newer AEDs are not significantly superior to traditional AEDs, they have different mechanisms and may be effective in cases where other first-generation drugs have failed [3]. A combination of traditional and newer AEDs is now being proposed to improve the efficacy and overall outcomes [4]. Perampanel (PER) is a selective and non-competitive alpha-amino-3-hydroxy-5-methyl-4-isoxazole-propionic acid (AMPA) receptor antagonist that selectively inhibits AMPA receptor-mediated synaptic excitation [5, 6]. PER can be administered once a day and has a long half-life of approximately 53–136 hours [7]. Additionally, PER may have the potential to control seizures that could not be controlled by earlier AEDs [8]. Epilepsy most commonly begins in childhood [9] and is often associated with cognitive and behavioral malfunction. PER has been widely investigated in different randomized controlled trials and clinical settings and is now reported as safe and effective [10]; however, very few studies have analyzed the application of PER among pediatric patients aged < 12 years. The present study aimed to evaluate the efficacy of PER as adjunctive therapy on pediatric focal epilepsies that were resistant to other drugs.

Seventy-one pediatric and adolescent patients aged ≤ 18 years (37 males and 34 females) who received PER treatment were diagnosed with focal epilepsy at Children’s Hospital, Zhejiang University School of Medicine from March 2020 to November 2020. Characteristics were observed, such as age, sex, epilepsy syndrome and etiology, personal history of AEDs, and clinical outcomes of seizure after follow-up for six months. The initial and maximum PER doses were adjusted based on the patient’s condition. Epilepsy ease include the general query “check author group and affiliation” in all articlesyndromes and seizure types were categorized in line with the criteria of the Commission on Classification and Terminology of the International League Against Epilepsy criteria (2017) [11]. The enrolled patients were diagnosed with epilepsy that was exhibited as focally originated seizures and/or focal discharges on electroencephalography. Epilepsy etiologies were divided into six categories: genetic, structural, immune, metabolic, infectious, and unknown. The reduction from baseline in seizure frequency was determined during the 6-month follow-up period to evaluate its efficacy. Patients whose seizure frequency was reduced by ≥ 50% or who were seizure-free were defined as responders, while a 100% reduction in seizure frequency was deemed seizure-free. SPSS Statistics V21.0 (IBM Corp., Armonk, NY, USA) was employed for statistical analysis [12]. Continuous variables are represented by means ± standard deviations, while categorical variables are represented by medians and interquartile intervals. Non-parametric tests were performed to compare the number of combined AEDs between responders and nonresponders. The Chi-square test was used to evaluate the sex difference in PER efficacy [13, 14]. *P* < 0.05 indicated statistical significance.

The median PER initiation age of all patients was 6 years (range: 5–9 years) (Table 1). The epilepsy categories were structural (*n* = 14), genetic (*n* = 12), metabolic (*n* = 1), infectious (*n* = 1), immunological (*n* = 3), and unknown (*n* = 40). Simultaneously, 19 of all the cases exhibited specific epilepsy syndromes, such as benign epilepsy with centrotemporal spikes (BECTS) (*n* = 8), West syndrome (*n* = 7), Dravet syndrome (*n* = 3), and Lennox–Gastaut syndrome (LGS, *n* = 1). The median age of the patients receiving combined anti-seizure treatments was 6 years (range: 5–9 years) at the PER initiation stage (Table 1). A 67.6% (48/71) overall response rate was achieved, while 33.8% (24/71) of the patients attained total seizure control. Additionally, 23 (32.4%) patients did not exhibit obvious alterations in seizure frequency. The inter-gender response rate (*P* = 0.61), PER initiation age (*P* = 0.38), combined AED number (*P* = 0.85), and etiology (*P* = 0.45) were not statistically significant (Table 2). A total of eight patients diagnosed with BECTS were responders after six months, with a seizure-free rate of 75% (6/8; Table 2). Electrical status epilepticus in sleep (ESES) cases showed a high response rate (77.7%, 7/9), while cases having Dravet syndrome attained a 66.7% (2/3) response rate. Seven patients diagnosed with West syndrome depicted a 28.6% (2/7) response rate, whereas three patients with tuberous sclerosis complex (TSC) appeared as nonresponders. All adverse effects occurred within the first six weeks of treatment, including nervousness, restlessness, behavioral deterioration, and fatigue, in ten patients (14.1%, 10/71) (Table 2). Eight patients started adverse events when taking 6.0 mg of PER, and all side effects improved when the dosage of PER was tapered down.

**Table 1:** Pediatric baseline and demographic characteristics (*N* = 71)

Variables	Values
Sex (female), *n* (%)	34 (47.9)
PER initiation age (y)	6 (5, 9)
Etiology, *n* (%)	
Structural	14 (19.7)
Genetic	12 (16.9)
Immune	3 (4.2)
Infectious	1 (1.4)
Metabolic	1 (1.4)
Unknown	40 (56.3)
Number of combination AEDs	3 (2, 3)
50% responders, *n* (%)	48 (67.6)
Seizure-free, *n* (%)	24 (33.8)
Specific epilepsy syndromes, *n*	
BECTS	8
West syndrome	7
Dravet syndrome	3
LGS	1
Side effects, *n*	
Nervousness	2
Restlessness	3
Behavioral deterioration	3
Fatigue	2

*PER* perampanel, *AEDs* antiepileptic drugs, *BECTS* benign epilepsy with centrotemporal spikes, *LGS* Lennox–Gastaut syndrome

**Table 2:** Demographic data and clinical results in responders compared with nonresponders

Factors	Responders (*n* = 48)	Non-responders (*n* = 23)	*P*
PER initiation age (y)	7.2 ± 3.5	5 (4, 9)	0.38
Number of combination AEDs	2.5 (2, 3)	3 (3, 3)	0.85
Female, *n* (%)	24 (50.0)	10 (43.5)	0.61
Etiology, *n* (%)			0.45
Genetic	5 (10.4)	7 (30.4)	
Infection	1 (2.1)	0 (0)	
Metabolic	1 (2.1)	0 (0)	
Structural	8 (16.7)	6 (26.1)	
Immune	2 (4.2)	1 (4.3)	
Unknown	31 (64.6)	9 (39.1)	
Specific epilepsy syndromes, *n*			
BECTS	8	0	
West syndrome	2	5	
Dravet syndrome	2	1	
LGS	1	0	

Responders and nonresponders referred to patients whose seizure frequency reduced by ≥ 50% and < 50% relative to baseline, respectively. *AEDs* antiepileptic drugs, *PER* perampanel, *BECTS* benign epilepsy with centrotemporal spikes, *LGS* Lennox–Gastaut syndrome

After an average 6-month follow-up, the response rate was 67.6%, while 33.8% became seizure-free. Our results were in accordance with those of previous studies conducted on pediatric patients aged > 12 years. According to previous studies on refractory epilepsy patients aged < 18 years, the responder rate was 31%–68%, while 9%–23% of patients achieved seizure freedom [15, 16]. A study comparing the efficacy of patients in < 12- and > 12-year groups showed that the difference in response rate was not statistically significant between the groups [17]. Our study stated a similar finding that age was not a significant factor in evaluating the efficacy of PER initiation time in responders and nonresponders (*P* = 0.38). A randomized study conducted on adolescents receiving PER and placebo displayed a statistically significant 50% response rate every 28 days in adolescent partial-seizure epilepsy treatment compared with placebo. The median seizure frequency reduction rate for PER was 58.0%, and that for placebo was 24.0%, demonstrating the efficacy of adjuvant PER for patients [5]. Another real-world pediatric case series in Taiwan of China stated that the seizure reduction rates of ≥ 50% were 44% and 31%, while the seizure-free rates were 13% and 10% at 6 and 12 months, respectively [18]. In our study, the characteristics of patients, including sex, etiology, PER initiation age, and the number of concomitant AEDs, showed no significant differences in therapeutic response. Although efficacy was not significantly different between genetic and non-genetic epilepsy in pediatric patients, a significantly improved 66.7% (2/3) response rate and seizure-free state in one case was attained in six months in cases with Dravet syndrome. A retrospective study analyzed the seizure frequency among ten cases with Dravet syndrome and described that five cases had a ≥ 50% reduction. The therapeutic effects of PER in diverse seizure subtypes are shown below: unilateral clonic type, 50% (3/6); myoclonic type, 33% (1/3); generalized tonic‒clonic type, 50% (4/8); atypical absence type, 33% (1/3); and focal impaired awareness type, 100% (1/1) [19]. The unicentric study conducted on 13 LGS cases demonstrated the high efficacy of PER adjuvant therapy by stating that 69.2% had seizure reduction rates of ≥ 50%, while 23.1% had seizure reduction rates of ≥ 75% at an average 10.8-month follow-up [20]. In our study, a single patient was diagnosed with LGS and was categorized as a responder after using PER for six months. Another European retrospective study on the pediatric population reported a 31% response rate and a 9% seizure-free rate after the first three months of therapy [21]. The study consisted of 58 patients (mean age: 10.5 years; range: 2–17 years) suffering from various refractory epilepsies, such as unclassified generalized epilepsy, focal seizures, West syndrome, LGS, and Dravet syndrome. Although with smaller sample sizes in diverse groups, analysis of seizure type, syndrome and etiology reported that patients with unknown etiology and structural-metabolic epilepsy with a response rate of 29% and 38%, respectively, showed superior outcomes to the genetic variant of epilepsy with a response rate of 18%. Correspondingly, cases with Dravet syndrome (50%), LGS (40%), focal epilepsy (33%), and unclassified generalized epilepsy (25%) displayed superior outcomes to cases with West syndrome (0%). Similarly, in our study, the outcome of cases with unknown etiology and structural epilepsy with response rates of 64.6% and 16.7%, respectively, showed superior outcomes to genetic epilepsy with a response rate of 10.4% in the responder group. Five of seven patients diagnosed with West syndrome were nonresponders. Some studies have also reported different PER response rates in pediatric TSC patients while demonstrating the efficacy of PER [15]. However, two of our cases responded poorly to PER treatment. BECTS is a common pediatric epilepsy disorder that begins from 3 to 13 years of age and has a nocturnal predominance. However, the therapeutic effects of PER on BECTS have rarely been reported until now. Although several previous studies consider BECTS to be a benign pediatric disorder that usually disappears at the age of 16 years, there might be atypical evolution of BECTS in some cases, resulting in syndromes such as Landau Kleffner syndrome, benign atypical partial epilepsy, and ESES. Our results revealed that all eight patients diagnosed with BECTS who showed ineffective treatment with one or more traditional AEDs were recorded as responders after six months. Simultaneously, a higher response rate can be achieved in ESES cases treated with PER. Thus, PER is extremely effective as an adjunctive therapy to treat BECTS and ESES patients. All adverse events occurred within two months of PER initiation, especially when taking 6.0 mg of PER, and the symptoms improved immediately when the dosage was tapered.

The limitations in our study were the small sample volume and the duration of follow-up. However, our study proved that PER was an effective medication for treating pediatric focal epilepsy, especially in BECTS/ESES patients.

## Data Availability

All data utilized in this work can be obtained from the corresponding author upon request.
